# Gene Polymorphisms Affecting the Pharmacokinetics and Pharmacodynamics of Donepezil Efficacy

**DOI:** 10.3389/fphar.2020.00934

**Published:** 2020-06-19

**Authors:** Jin Lu, Xiuzhe Wang, Lili Wan, Jianliang Fu, Yan Huo, Yuwu Zhao, Cheng Guo

**Affiliations:** ^1^Department of Pharmacy, Shanghai Jiao Tong University Affiliated Sixth People's Hospital, Shanghai, China; ^2^Department of Neurology, Shanghai Jiao Tong University Affiliated Sixth People's Hospital, Shanghai, China; ^3^School of Medicine, Shanghai Jiao Tong University, Shanghai, China

**Keywords:** donepezil, clinical efficacy, gene polymorphisms, pharmacogenetics, pharmacodynamics

## Abstract

Donepezil (DNP) is the first-line drug used for Alzheimer's disease (AD). However, the therapeutic response rate of patients to DNP varies from 20 to 60%. The main reason for the large differences in the clinical efficacy of DNP therapy is genetic factors, some of which affect pharmacokinetics (PK), while others affect pharmacodynamics (PD). Thus, much emphasis has been placed on the investigation of an association between PK- and PD-related gene polymorphisms and therapeutic response to DNP, but a consistent view does not yet exist. In this review, we summarize recent findings regarding genetic factors influencing the clinical efficacy of DNP, including substantial differences in individual responses as a consequence of polymorphisms in Cytochrome P450 (CYP) 2D6, CY3A4, CY3A5, APOE, ABCA1, ABCB1, ESR1, BCHE, PON-1, CHRNA7, and CHAT. We also discuss possible strategies for the evaluation of the clinical efficacy of DNP, with a specific focus on possible biomarkers of PK/PD parameters, and provide perspectives and limitations within the field, which will also be beneficial for understanding the multiple mechanisms of DNP therapy in AD.

## Introduction

Donepezil (DNP) is a cholinesterase inhibitor widely used for the treatment of mild to moderate Alzheimer's disease (AD) in the past 20 years. Recently, an increasing number of randomized case–control studies have confirmed the clinical value of DNP in the treatment of mild to moderate Alzheimer's disease (Cacabelos et al., 2016; Birks and Harvey, 2018). Due to its good liposolubility, it can easily pass through the blood–brain barrier. DNP binds to cholinesterases and has a strong affinity for acetylcholinesterase, especially in the cerebral cortex (Prvulovic and Schneider, 2014). Thus, DNP has the beneficial therapeutic effect of inhibiting acetylcholinesterase in the brain and few adverse effects (which are mainly gastrointestinal reactions) (Noetzli et al., 2014). It is the first-line treatment for mild to moderate AD in more than 75 countries worldwide (Birks and Harvey, 2018).

However, the clinical response of AD patients to DNP varies largely, and the therapeutic efficacy ranges from 20 to 60% [5–9] (Matsui et al., 1999; Raskind et al., 2000; Yang et al., 2011; Albani et al., 2012; Barth et al., 2012). Pharmacogenetic factors account for 60–90% of drug variability in drug disposition and pharmacodynamics (Cacabelos, 2008). A large number of related studies have shown that the main reason for the large differences in the clinical efficacy of DNP therapy may be closely related to genetic factors (Raskind et al., 2000; Noetzli et al., 2014). In this review, we attempt to (1) summarize the genetic variants that may impact the response to DNP treatment in AD patients according to pharmacogenetic and pharmacodynamic effects as well as (2) provide an overview of possible PK/PD biomarkers of DNP efficacy and perspectives and limitations within the field.

## Data Sources and Search Strategy

A comprehensive search of studies about genes related to the pharmacokinetics (PK) and pharmacodynamics (PD) of DNP treatment in AD patients published up to April 2020 was performed. Publications were restricted to the English language, and well-designed studies were included. Studies were identified through an electronic search of two databases: PubMed and Web of Science. For the search strategy, we used the subject words “donepezil”, “clinical efficacy”, “gene polymorphisms”, “pharmacogenetics”, “pharmacodynamics”, and “Alzheimer's disease” and their free words to search the two databases for articles written in English. Relevant reference lists were also searched. We included studies involving quantitative analysis if they met the following criteria: (1) research papers of randomized case–control studies considering the association between gene polymorphism and efficacy of DNP in AD; (2) studies reporting sufficient information on inclusion criteria and exclusion criteria for patients; and (3) studies reporting the numbers of enrolled patients and genotype frequencies in patients. We excluded (1) duplicates within and between the databases, (2) studies of animals, (3) studies not related to dementia of AD, (4) studies with no analysis of donepezil when it was used as the basic treatment or a positive control, and (5) articles that were not research papers, such as letters to the editor, case reports, or review articles. Studies used to analyze the efficacy of donepezil in patients with AD mostly included Caucasian and Mongoloid populations.

## Efficacy-Related Pharmacokinetic Genes

Metabolism is one of the major causes leading to variability in the clinical response to DNP (Rogers and Friedhoff, 1996; Winblad et al., 2001). DNP is mainly metabolized by hepatic enzymes, and 6-deoxy-DNP (6-O-DNP) is the main active metabolite (Tiseo et al., 1998; Cascorbi, 2003; Suh et al., 2005; Prvulovic and Schneider, 2014; Adlimoghaddam et al., 2018). Cytochrome P450 (CYP) 2D6, CYP3A4, CYP3A5, and CYP2C9 are thought to be involved in the metabolism of DNP (Noetzli et al., 2014). It has been reported that donepezil is mainly metabolized by CYP2D6 and CYP3A4 in the liver (Noetzli and Eap, 2013); thus, in recent years, a number of studies have reported the association between CYP2D6 and CYP3A4 polymorphisms and the clinical efficacy of DNP, which we will elaborate in the following paragraphs.

### CYP3A4 and CYP3A5

CYP3A4 and CYP3A5 are genes encoding metabolic enzymes related to the efficacy of DNP in AD patients (McEneny-King et al., 2015). Italian scholar Laura Magliulo et al. studied the clinical effects of the CYP3A genes on DNP efficacy in 54 AD patients and 285 control patients in Italy. They found that the genetic polymorphisms in CYP3A4 and CYP3A5 did not significantly affect DNP metabolism and patients. However, AD patients with the CYP3A5*1 allele had better clinical outcomes than patients with the CYP3A5*3/*3 allele, but the results were not significant (Magliulo et al., 2011). Another study carried out in Chinese patients with AD also indicated that the CYP3A4 gene does not influence the efficacy of DNP (Ma et al., 2019).

The CYP3A allele does not affect the pharmacokinetics of DNP *in vivo*, which may be the reason why the CYP3A allele is not significantly associated with the efficacy of DNP. We demonstrated that CYP3A4 contributes much less to the metabolism of DNP *in vivo*, while CYP2D6 mostly contributes to the metabolism of DNP (Lu et al., 2015). Our findings were also confirmed by the Swiss team Muriel Noetzli and colleagues. They studied the effect of CYP3A on DNP clearance in patients. Among the 129 Swiss AD patients, there were 5 CYP3A variants: CYP3A4*1B (rs2740574), CYP3A4 (rs4646437), CYP3A4*22 (rs35599367), CYP3A5*3 (rs776746), and CYP3A7*1C (−262T > A and −270T > G), and these variants did not affect the pharmacokinetics of DNP *in vivo* (Noetzli et al., 2014).

### CYP2D6

#### CYP2D6 Is the Main Enzyme Metabolizing DNP

Orally administered DNP has an approximately 95% plasma protein binding rate (Adlimoghaddam et al., 2018). After oral administration of DNP, it is metabolized by the liver P450 enzymes, and 6-O-DNP is the most active metabolite (Matsui et al., 1999; Pilli et al., 2011; Barth et al., 2012). Lu et al. performed an *in vivo* study and identified CYP2D6 as the predominant metabolic enzyme of DNP (Noetzli et al., 2014; Lu et al., 2015). Data show that CYP2D6 is involved in more than 25% of drug metabolism, and this gene has more than 90 allelic variations (Cascorbi, 2003). CYP2D6 gene polymorphisms affect the efficacy of DNP in AD patients, who either experience a therapeutic effect from DNP at the prescribed drug dose or no response to DNP (Honghao, 2013). The mechanism may involve the association between CYP2D6 gene polymorphisms and the plasma concentration of DNP. Studies have been conducted on the relationship between CYP2D6 gene polymorphism, DNP plasma concentration, and effect of DNP in Caucasian and Mongoloid populations.

#### The Association Between CYP2D6 Polymorphisms and the Efficacy of DNP

CYP2D6 rs1080985 is the main mutation in the Caucasian population. CYP2D6 rs1080985 is the CYP2D6*2A variant, which confers a normal phenotype to Caucasian people. Studies have reported CYP2D6*2A (rs1080985) polymorphism influences the clinical efficacy of DNP. Studies have explored the relationship between the rs1080985 polymorphism and the efficacy of DNP; however, the results are not consistent.

Alberto Pilotto et al. studied 127 patients with Alzheimer's disease in Italy (Pilotto et al., 2009). It has been reported that there is an association between the CYP2D6*2A (rs1080985) G allele and patient responses to DNP. The rs1080985 G allele is associated with a faster rate of drug metabolism, resulting in DNP being less effective in patients (Zanger et al., 2001; Gaedigk et al., 2003), and Alberto Pilotto's study confirms this conclusion from a clinical perspective (Pilotto et al., 2009). Diego Albani et al. studied 415 patients with Alzheimer's disease in Italy (Albani et al., 2012). By using logical linear regression analysis, the rs1080985 G allele was indeed associated with an ineffective therapeutic effect of DNP.

Muriel Noetzli et al. believe that different alleles of CYP2D6 influence the metabolic behavior of DNP, which may be the main reason for the differences in DNP treatment efficacy in AD patients. The CYP2D6 gene polymorphism caused a difference in the clearance rate of DNP in patients. In this study, 129 AD patients treated with DNP therapy were enrolled, and the researchers genotyped the relevant CYP2D6*3, CYP2D6*4, CYP2D6*5, and CYP2D6*6 alleles in patients. The researchers obtained the pharmacokinetic parameters of DNP *in vivo* by establishing a population pharmacokinetic model. The results showed that the CYP2D6 alleles had different effects on the clearance of DNP *in vivo*. Poor metabolizers had a 32% slower rate of *in vivo* clearance of DNP and a 67% slower metabolism rate than ultra-rapid metabolizers (Noetzli et al., 2014). The CYP2D6 gene polymorphism affects the metabolic behavior of DNP in patients, which may lead to inconsistencies in the efficacy of DNP.

At the same time, studies have found that the metabolic behavior of CYP2D6 *in vivo* can be changed. R. Cacabelos et al. simultaneously analyzed the APOE and CYP2D6 genes and found that patients with the APOE4/4 genotype may be complete metabolizers of CYP2D6*1/*1, suggesting that patients with a homozygous APOE-4 have highly potent CYP2D6 drug metabolism (Cacabelos and Martinez-Bouza, 2011). Similarly, the distributions of the APOE-4/4 gene in extensive metabolizers and poor metabolizers (as dichotomized based on CYP2D6) were also different (Carson et al., 2008). However, it is not clear whether the effects of APOE polymorphism on the CYP2D6 gene can influence DNP efficacy. Lu et al. reported a trend toward a combined effect of APOE and the CYP2D6 rs1065852 polymorphisms on the clinical efficacy of DNP in Han Chinese patients with AD. The study identified that the patients who were APOE E3 noncarriers and who had the CYP2D6*10/*10 allele showed the best clinical response to DNP (Lu et al., 2016), and the authors believe the mechanism may be related to the effect of APOE on P450-related enzymes (Lu et al., 2016).

However, in Asians rs1065852 polymorphism is the most common mutant allele: it is reported that 37.9% of the Chinese population carries the CYP2D6*1 variant, 51.3% carries the CYP2D6*10 variant (Sakuyama et al., 2008; Saito et al., 2018). For the CYP2D6 rs1065852 polymorphism, Yuan Zhong et al. investigated the relationship of the CYP2D6*1/*1, CYP2D6*1/*10, and CYP2D6*10/*10 alleles in 106 Asian patients with mild to moderate Alzheimer's disease with DNP efficacy. The study found that patients with the CYP2D6*10/*10 allele had better efficacy than those with the other CYP2D6 genotypes, and the steady-state plasma concentration (Cp/dose) of DNP in patients with the CYP2D6*10/*10 allele was significantly higher than those of the other two groups. It is predicted that the peripheral blood concentration may also be a factor related to the efficacy of DNP treatment. This study was limited by the number of samples, and a larger sample size will be needed in the future (Zhong et al., 2013). Thitipon Yaowaluk et al. also reported that CYP2D6*10 carriers have a better therapeutic response to DNP than patients with other CYP2D6 genotypes because they have a higher Css of DNP (Yaowaluk et al., 2019).

However, other studies reported that the efficacy of DNP was influenced by the concentration of DNP but not by CYP2D6 polymorphisms. Miranda, L F et al. followed patients for 12 months and found that a good response was influenced by the concentration of DNP, which was associated with efficacy (Miranda et al., 2017).

On the other hand, there are studies reporting that the correlation between the rs1080985 G allele and efficacy is not significant. Aleksandra Klimkowicz Mrowiec et al. studied 88 Caucasian patients who received DNP for 10 months and concluded that the GG, CG, and CC alleles of rs1080985 were not associated with the efficacy of DNP (Pilotto et al., 2009).

#### Discussion of CYP2D6 and the Efficacy of DNP

CYP2D6 (rs1065852), CYP2D6 (rs1080985), CYP2D6*3 (rs35742686, 2549delA, P/N: 4312554), CYP2D6*4 (rs3892097, 1846G > A, P/N: 4312555), CYP2D6*6 (rs5030655, 1707delT, P/N: 4312556), CYP3A4*1B (rs2740574, −392A > G), and CYP2D6*10 have been studied for their association with DNP efficacy. However, the results are not consistent. We believe the main reasons for the inconsistent results include the following: 1) these studies were conducted in different ethnic groups. Research in different ethnic groups will have bias in enrollment, for example, most studies are not multicenter for collection of samples; in addition, the distribution frequency of genes in different ethnic groups is different, leading to different statistically significant results; and part of the reason may be: 2) they investigated concentrations of racemic DNP as opposed to S-DNP, which is the pharmaco-effective enantiomer of DNP. Thus, the inconsistency may have resulted from the use of racemic-DNP and the differences in the metabolism of each enantiomer, including the effective S-enantiomer, in the liver, resulting in the Cp/dose of racemic DNP being unable to be used to evaluate the clinical outcome of the drug.

Based on the above findings, our group has focused on the efficacy of S-DNP. We have shown that CYP2D6*1/*10 and CYP2D6*10/*10 (rs1065852) are the two alleles with the highest mutation frequency in Han Chinese populations (Lu et al., 2016). We further explored whether the plasma concentrations of S-DNP (based on CYP2D6 polymorphisms) were significantly associated with therapeutic responses. The findings suggest that plasma concentrations of S-DNP influence the therapeutic outcomes following treatment with DNP in Han Chinese patients with Alzheimer's disease. Therefore, the results suggest that determining a patient's steady-state plasma concentration of S-DNP in combination with their CYP2D6 genotype might be useful for clinically monitoring the therapeutic efficacy of DNP (Lu et al., 2015; Lu et al., 2016) and further exploring the association of CYP2D6 and APOE. We confirmed that both CYP2D6 and APOE have an influence on therapeutic response to DNP (Lu et al., 2016). Our study may explain the inconsistent results of other studies.

CYP2D6 may be an important genetic marker for the clinical efficacy of DNP.

The plasma concentration of S-DNP is strongly associated with DNP efficacy. However, studies aimed at specific populations with larger samples will be needed in the future to confirm this conclusion.

### CYP2C9

Laura Magliulo et al. studied the clinical effects of the CYP2C9 gene on DNP efficacy in 54 AD patients and 285 control patients in Italy. They found that genetic polymorphisms of CYP2C9 did not significantly affect DNP metabolism and patients (Magliulo et al., 2011). These results are consistent with Lu's study, which identified that CYP2C9 contributes little to the metabolism of DNP by exploring the kinetic parameters of the 6-ODD metabolite of DNP from the perspective of cDNA-expressed P450 enyzmes (Lu et al., 2015).

### ABCB1

ABCB1, which regulates the movement of compounds across the blood–brain barrier (BBB), may influence the transport of DNP (McEneny-King et al., 2015). Some studies have focused on the association between ABCB1 polymorphisms and the efficacy of DNP, and the results suggest that ABCB1 polymorphism is not one of the main reasons for the variations in the therapeutic response to DNP. Muriel et al. reported that in a study of a total of 129 patients, no association was found between ABCB1 polymorphism and DNP efficacy (Noetzli et al., 2014). Laura Magliulo et al. reported that ABCB1 (3435C > T, 2677G > T/A, and 1236C > T) polymorphisms had no impact on the clinical outcome in 54 Italian patients (Magliulo et al., 2011). The same conclusion was obtained by Thitipon Yaowaluk et al. in patients in Thailand. No significant association of ABCB1 3435C > T or ABCB1 1236C > T with the Cp of DNP or the clinical efficacy of DNP was found in this study (Yaowaluk et al., 2019).

We determined that both (R)- and (S)-DNP were not P-gp substrates (Lili et al., 2013), which may be the reason for the negative outcome of DNP treatment in patients with ABCB1 polymorphisms. McEneny-King et al. also reported that DNP is not a substrate of P-gp but a weak inhibitor of DNP (McEneny-King et al., 2015), which is in agreement with our previous report.

## Pharmacodynamic-Related Genes

Studies have shown that DNP efficacy is influenced by polymorphisms in pharmacodynamic genes. Apolipoprotein E (APOE), which is believed to be associated with AD pathogenesis, has been reported to modulate the response to DNP treatment; ABCA1, which plays a key role in cholesterol transport and APOE metabolism in the brain, has been reported to be related to Alzheimer's disease. Thus, some studies have focused on the association between ABCA1 polymorphisms and DNP efficacy (**Table 1**).

Since DNP functions as an acetylcholinesterase inhibitor, the related BCHE, PON-1, CHRNA 7, and ChAT polymorphisms have been well studied. Due to sex differences, the ESR1 gene is another popular topic of related studies (**Table 1**).

**Table 1:** Differential relationships between DNP efficacy and the related PK/PD gene polymorphisms in patients with Alzheimer’s disease in different populations.

Genes	polymorphisms	Association/no association and Population	number of patients	Scale type	Follow-up time period	references
CYP2D6	CYP2D6 (rs1065852)	Y,Chinese;	77;96;85	MMSE;MMSE;TMSE	3m;6m;36m	Zhong et al., 2013; Lu et al., 2015; Yaowaluk et al., 2019
	CYP2D6 (rs1080985)	Y,Italian;American;German	415;115;203	MMSE;MMSE;/	6m;6m;/	Gaedigk et al., 2003; Pilotto et al., 2009; Albani et al., 2012
	CYP2D6*3 (rs35742686, 2549delA, P/N: 4312554), CYP2D6*4 (rs3892097, 1846G > A, P/N: 4312555), CYP2D6*6 (rs5030655, 1707delT, P/N: 4312556), CYP3A4*1B (rs2740574, −392A > G)	Y,Swiss	129	/	1–96m	Noetzli et al., 2014
	CYP2D6*3	N,Italian	92	MMSE	12m	Chianella et al., 2011
	CYP2D6 (rs1080985)	N,Polish	116	MMSE,CDT,IADL	1m	Klimkowicz-Mrowiec et al., 2013
CYP3A4	CYP3A4*1B(rs2740574);CYP3A4*3(rs4986910),CYP3A4*4(C:30634211_30)	N,Italian	42	MMSE,CDR,ADL,CIBIC-plus	3m	Magliulo et al., 2011
	CYP3A4*1B (rs2740574), CYP3A4 (rs4646437), CYP3A4*22 (rs35599367),	N,Swiss	129	/	1m-96m	Noetzli et al., 2014
CYP3A5	CYP3A5*3 (rs776746)	N,Swiss; N,Thailand	129;85	/;TMSE	1m-96m	Noetzli et al., 2014; Yaowaluk et al., 2019
	CYP3A5*1,CYP3A5*2(C:30633862_10);CYP3A5*6(C:30203950_10);	N,Italian	42	MMSE,CDR,ADL,CIBIC-plus	3m	Magliulo et al., 2011;
CYP2C9	CYP2C9 (rs1057910 and rs4918758),	N,Chinese	179	CDR,ADAS-cog,MMSE	12m	Ma et al., 2019
ABCB1	ABCB1 2677G > T (rs2032582), ABCB1 3435C > T (rs1045642), ABCB1 1236C > T (rs1128503)	N,Swiss	129	/	1m-96m	Noetzli et al., 2014
	ABCB1(1236C > T,3435C > T,2677G > A/T)	N,Italian;N,Thailand	42;85	MMSE,CDR,ADL,CIBIC-plus;TMSE	3m;36m	Magliulo et al., 2011; Yaowaluk et al., 2019
	ABCB1(rs1045642,rs2032582,rs1128503)	N,Chinese	88	MMSE	3m	Lu et al., (data not shown)
ABCA1	ABCA1(rs2230806)	Y,Chinese	88	MMSE	3m	Lu et al., 2018
	ABCA1 (rs2230808)	N,Chinese	88	MMSE	3m	Lu et al., 2018
APOE	APOE E4 carriers have the best response to DNP	Iberian;Italian;Korean	155;81;51	MMSE;MMSE;ADAS-cog	12m;12–16m;12m	Bizzarro et al., 2005; Choi et al., 2008; Cacabelos and Martinez-Bouza, 2011
	APOE E4 noncarriers have the best response to DNP	Italian;Canadian	25;40	MMSE;ADAS-cog,MMSE	1m;8m	Poirier et al., 1995; Borroni et al., 2002
	APOE E4 carriers have no response to DNP	Chinese;Italian;Janpanese;American;Thailand	96; 115; 171;61;938; 165; 85	MMSE;MMSE; MMSE; MMSE,HDS-R; MMSE,ADAS-Cog;ADAS-Cog;TMSE	6m; 6m; 12m;1-6m;9m;3m;36m	Pilotto et al., 2009; Nozawa et al., 2009; Santoro et al., 2010; Chianella et al., 2011; Zhong et al., 2013; Waring et al., 2015; Yaowaluk et al., 2019
	APOE E3 noncarriers have the best response to DNP	Chinese	85	MMSE	3m	Lu et al., 2016
ESR1	ESR1	Y,Italian	157	MMSE	15m	Scacchi et al., 2014
BCHE	BCHE(rs1803274, rs1355534, rs1803274)	N,Italian;Spainish	101; 114	MMSE; SIB,ADCS-ADL	15m; 24m	Blesa et al., 2006; Scacchi et al., 2009
	BCHE (rs1803274)	Y, American	145	MMSE	36m	Sokolow et al., 2017
		N,Italian	92	MMSE	12m	Chianella et al., 2011
PON-1	PON-1	Y,Italian	42	MMSE	9m	Pola et al., 2005
CHRNA7	CHRNA7 (rs8024987)	Y,Chinese Taiwanese;	204	MMSE	6m	Weng et al., 2013;
	CHRNA7 (rs6494223)	Y/N,Brazilian	177	MMSE	6m	Braga et al., 2015
ChAT	ChAT (rs2177369)	Y,Italian	101	MMSE	15m	Scacchi et al., 2009

### APOE

Apolipoprotein E (APOE) is a polymorphic protein with three alleles: E2, E3, and E4. APOE is mainly involved in the transformation and metabolism of lipoproteins (Uddin et al., 2019). In recent years, several studies have shown that APOE polymorphism is associated with the efficacy of DNP in the treatment of Alzheimer's disease.

It is widely believed that the E4 allele is a “risk factor” for AD, and patients with at least one E4 allele in the APOE gene are defined as carriers of the E4 gene (Josefsson et al., 2017). In the study of the relationship between DNP efficacy and the APOE gene, the results have been inconsistent.

(1) Some studies have shown that patients with AD carrying the E4 allele have the best DNP efficacy (Bizzarro et al., 2005; Choi et al., 2008; Cacabelos and Martinez-Bouza, 2011); (2) other studies have shown that E4 noncarriers responded better to DNP than E4 carriers (Poirier et al., 1995; Borroni et al., 2002). In addition, (3) some studies have shown that APOE E4 has no impact on DNP efficacy (Nozawa et al., 2009; Pilotto et al., 2009; Santoro et al., 2010; Chianella et al., 2011; Zhong et al., 2013; Waring et al., 2015; Yaowaluk et al., 2019). (4) We reported a significant difference in the frequency of APOE E3 alleles between DNP responders and nonresponders: E3 noncarriers showed a better response to DNP treatment than E3 carriers; however, we did not find a significant difference in APOE E4 frequency between responders and nonresponders (Lu et al., 2016). This may partially result from the differential A*β* peptide production, which is associated with the APOE E2 and E4 (the alleles in E3 noncarriers), between carriers of different alleles, which may be compensated for by DNP-induced sAPP production (Choi et al., 2008; Xiao et al., 2016).

Moreover, the combined effects of APOE and CYP2D6 genotype on DNP efficacy have been reported. In one mechanism, the APOE-related DNP response involves CYP2D6-related effects on liver metabolism (Lu et al., 2016). APOE-CYP2D6 interactions might influence the therapeutic response in AD *via* changes in lipid metabolism and liver function (Cacabelos and Martinez-Bouza, 2011).

### ABCA1

ABCA1 is a cholesterol transporter that neutralizes the A*β* aggregation capacity in an APOE-dependent manner. ABCA1 enables the clearance of amyloid *β* (A*β*) peptide from the brain in mouse models through its role in the lipidation of APOE. DNP treatment reduced cholesterol accumulation in adult neural stem cells *in vitro*. ABCA1 gene polymorphisms may influence the efficacy of DNP (Lu et al., 2018).

There are few studies on ABCA1, but one of our studies has reported the association between ABCA1 and the efficacy of DNP: patients with the ABCA1 rs2230806 GG genotype responded better to DNP treatment than those with the AA and AG genotypes. We consider that the probable reasons for the ABCA1 rs2230806 genotype influencing DNP efficacy may be the result of DNP-induced sAPP production (Choi et al., 2008). Other probable reasons may include the following: 1) DNP has been shown to induce sAPP production (Mori et al., 1995; Choi et al., 2008). 2) ABCA1 works as a transporter that transports A*β* from the brain into the blood, eventually causing A*β* to be cleared from the brain and reducing the level of A*β* in the brain. The mechanism may be related to ABCA1's ability to reduce *β*-secretase activity. In addition, ABCA1 promotes cholesterol efflux to the cerebrospinal fluid, thereby improving cognitive decline in AD patients (Yassine et al., 2016; Marchi et al., 2019). These mechanisms suggest that ABCA1 can reduce the production of A*β* by regulating cholesterol efflux and reducing the intracellular content of cholesterol, thereby improving cognitive decline, possibly in an APOE-dependent manner, while the cholesterol transporter ABCA1 neutralizes the A*β* aggregation capacity in an APOE-dependent manner (Lupton et al., 2014). 3) Based on the above analysis, ABCA1 influences DNP efficacy *via* A*β* aggregation, but this mechanism requires further study.

We also found that patients who were APOE E3 noncarriers and had the ABCA1 rs2230806 GG genotype tended to have a better clinical response to DNP therapy than other patients, which indicated that there may be crosstalk between APOE E3 and ABCA1. The transcription of APOE is regulated by LXR-*α*, and the expression of ABCA1 mRNA is regulated by LXR-*α* and increases in parallel with APOE transcription during apoptosis (Cacabelos, 2008), suggesting a potential mechanism.

### ESR1

The gene encoding estrogen receptor alpha (ER*α*) is reported to be involved in cognitive function. One of the potential mechanisms by which estrogen modulates cognitive function is *via* the cholinergic system (Tinkler and Voytko, 2005).

Some studies have suggested that gene polymorphism in estrogen receptor alpha (ESR1) is related to the efficacy of DNP. Animal experiments have shown that the cholinergic system may be regulated by estrogen and that estrogen affects cognitive ability (Tinkler and Voytko, 2005). Some researchers have studied the association between AD and ESR1 (the gene encoding the ER gene) (Corbo et al., 2006; Sundermann et al., 2010). However, whether genetic variation of ESR1 plays a role in drug response in AD has not been studied thus far.

A study by Renato Scacchi et al. examined whether ESR gene polymorphisms affect the therapeutic effects of acetylcholinesterase inhibitors. There are two variant sites for ESR1: rs2234693 and rs9340799. The allelic types are PPXX, PPXx, PpXX, and PpXx. A total of 184 Caucasians participated in one study. The study found that PX carriers had a higher drug response to DNP than noncarriers (Scacchi et al., 2014).

The study also found that women were more sensitive to DNP treatment than men. Since estrogen may affect the biosynthesis of acetylcholine, it mainly functions *via* ERa by regulating the activity of acetyltransferases. This may be the reason why women are more sensitive to DNP treatment. At the same time, the *in vitro* study showed that patients carrying the P allele had increased transcription of ESR1 and thus the activity of estrogen compared with patients carrying other alleles. This has also been confirmed by other clinical trials: in menopausal women carrying the Px allele, the concentration of estradiol in plasma was higher than that in women carrying other alleles(Scacchi et al., 2014). The P and X alleles in ESR1 promote the biosynthesis of acetylcholine, thereby enhancing the inhibition of drug-related acetylcholinesterase. Thus, the total amount of effective acetylcholinesterase is increased, and the effect of cognitive reduction is reduced (Scacchi et al., 2014).

### BCHE

Butyrylcholinesterase (BCHE) belongs to the cholinergic enzyme family. One single nucleotide polymorphism generally reported is BChE rs1803274 (the so-called K allele), and another is BChE rs1355534. The correlation between the K variant and AD has been extensively studied, and several studies have performed case–control comparisons. However, the results are not consistent. Lehmann et al. concluded that there was no significant association between the K variant and the onset of AD, and the K variant was not a risk factor for AD. However, their substudy showed a significant increase in the risk of AD in the group with men over the age of 75 who carried the K and E4 genes compared with the control group (Lehmann et al., 2001).

Sophie Sokolow et al. reported that BChE rs1803274 (K allele) is associated with a poor response to donepezil therapy after a 3-year observation in 145 patients with MCI (Caucasian), which indicated that BChE rs1803274 (K allele) may be a genetic marker of donepezil efficacy (Sokolow et al., 2017). However, Italian scholar Renato Scacchi et al. studied the efficacy of DNP in patients with BChE rs1355534 and BChE rs1803274 (K allele) and delayed-onset AD, and they concluded that there was no significant association between the BChE gene and DNP efficacy (Scacchi et al., 2009). Similar results were reported by Blesa et al., who studied the efficacy of DNP and Lismin in the treatment of AD patients and the relationship between the K allele and rs1803274 allele. They did not find a statistically significant difference (Blesa et al., 2006).

De Beaumont L et al. reported that carriers of the APOE E4 and/BCHE-K∗ variants responded better to donepezil therapy than other patients after a three-year observation. They reported that APOE E4- and BCHE-K∗-positive subjects had reduced brain cholinergic activity, which may be the reason for their better response to donepezil therapy (De Beaumont et al., 2016).

### PON-1

Paraoxonase (PON-1) is a versatile biologically active arylesterase that hydrolyzes surrounding neurotoxins. In addition, it is also a potent exogenous acetylcholinesterase inhibitor (Kondo and Yamamoto, 1998; Costa et al., 2005).

Roberto Pola et al., from Italy, explored the relationship between genetic polymorphisms and the efficacy of acetylcholinesterase inhibitors (DNP and rivastigmin) in AD patients. QQ, QR, and RR are three alleles of the 192 site of the PON-1 gene. The responsive group had a significantly higher frequency of the R allele than the unresponsive group, which suggests that the 192 Q/R gene polymorphism of PON-1 affects the efficacy of acetylcholinesterase inhibitors in patients. A total of 73 Brazilian AD patients were enrolled in the study. Among all of the patients taking acetylcholinesterase inhibitors, the proportion of patients who carried the R genotype in the group with superior efficacy was significantly higher than that in the ineffective group. There were no significant differences in DNP efficacy between the rivastigmin and other treatment groups. Studies have shown that AD patients with the PON-1 gene carrying the R allele are more susceptible to treatment with acetylcholinesterase inhibitors than AD patients with the QQ allele (Pola et al., 2005).

### CHRNA7

Acetylcholine receptor subunit *α*7 (CHRNA7) plays a role in the pathogenesis and prevention of AD. There are a few studies on whether the CHRNA7 gene polymorphism affects the efficacy of DNP in patients with AD. Researchers in Brazil posited that the CHRNA7 gene polymorphism affects the efficacy of DNP in patients with AD. The researchers followed up patients for 2 years to explore the association between the efficacy of acetylcholinesterase inhibitors and the T allele of rs6494223. After 6 months of observation, in 77 patients receiving DNP, there was a significant association between the T allele of CHRNA7 and the efficacy of acetylcholinesterase inhibitors in patients with MMSE >20. However, after 24 months of treatment, the T allele of CHRNA7 was not significantly associated with treatment efficacy (Braga et al., 2015).

CHRNA7 gene polymorphism has been thought to be associated with schizophrenia and AD (Joo et al., 2010; Ancin et al., 2011). The T allele of rs6494223 is associated with a progressive decrease in mild cognitive decline and a reduction in mental disorder syndrome (Carson et al., 2008). The T allele may be indicative of deeper choline dysfunction, confusion, and a better response to acetylcholinesterase inhibitors. This hypothesis has been observed in patients with dementia caused by dementia with Lewy bodies and Parkinson's disease (Court et al., 2001). One Brazilian study was the first to study the efficacy of acetylcholinesterase inhibitors in patients with AD (Braga et al., 2015).

Another study by Chinese scholars in Taiwan concluded that female AD patients with the rs8024987 allele had better efficacy with acetylcholinesterase inhibitors than male patients with this allele. These carriers have better efficacy with galantamine than noncarriers who use DNP (Weng et al., 2013).

A probable mechanism is that acetylcholinesterase inhibitors increase the concentration of acetylcholine, which binds to a7 nAChR, encoded by CHRNA7. The effect of CHRNA7 polymorphism on the effects on cognitive function induced by acetylcholine inhibitors in humans may be accomplished by the following: 1. regulation of the release of presynaptic neurotransmitters; 2. enhancement of memory function *via* regulation of cholinergic neurotransmission; 3. neuroprotection *via* a7 nAChR; 4. upregulation of a7 nAChR by an acetylcholinesterase inhibitor; and 5. positive allosteric regulation of a7 nAChR associated with galantamine (Weng et al., 2013).

Taken together, these results show that CHRNA7 gene polymorphism may be one of the genetic markers for the efficacy of DNP therapy.

### ChAT

Choline acetyltransferase is encoded by the ChAT gene located on chromosome 10q 11.2 (Francis et al., 1999; Li et al., 2012). The ChAT rs2177369 polymorphism plays an important role in the formation of acetylcholine. DNP works on the cholinergic system, and thus, it is thought to be related to variability in drug efficacy. Italian scholar Renato Scacchi et al. studied the association between the ChAT rs2177369 polymorphism and the efficacy of DNP in the treatment of late-onset AD. Their study concluded that the G/G genotype was considered a risk gene relative to the G/A+A/A gene. Eighty-seven patients (27.7% males, 72.3% females; age range 56–93 years) took a daily dose of 5 mg DNP, and 14 patients took a daily dose of 10 mg DNP. The ChAT rs2177369 polymorphism was analyzed. The study showed that compared with patients with AD with the G/A+A/A genotypes, AD patients carrying the ChAT rs2177369 G/G genotype had a poorer response to DNP, suggesting that the ChAT gene is a risk gene (Scacchi et al., 2009).

## Conclusions and Future Perspectives

DNP plays an important role in the treatment of Alzheimer's disease, but its individual efficacy varies widely, leading to treatment failure and economic waste in clinical therapy (Francis et al., 1999). Gene polymorphisms affect the pharmacokinetics and pharmacodynamics of donepezil efficacy. Therefore, genetic factors are closely related to individual variations in efficacy. The discovery and development of genetic biomarkers provide individualized medicine based on a patient's genetic markers. Studies on the efficacy of DNP and related gene polymorphisms in various ethnic groups and various countries are summarized above. To our knowledge, this is the first review which summarized gene polymorphisms which affect the pharmacokinetics and pharmacodynamics of donepezil efficacy, providing new ideas and new targets for the DNP treatment of AD.

Among the analyses we mentioned, CYP2D6 and APOE genes were the most explored genes. CYP2D6 polymorphisms certainly influence the efficacy of DNP among different people. According to current studies, CYP3A4, CYP3A5, and ABCB1 have no significant influence on DNP efficacy. However, with regard to CYP2D6, ABCA1, APOE, ESR1, BCHE, PON-1, CHRNA7 and CHAT and their mechanism, further research is needed. Additionally, it is noteworthy that variations in a single gene probably have a limited impact on drug efficacy; multiple gene variations may have a greater impact on drug efficacy. There are some reports that have studied the combined effect of two genes on DNP efficacy, such as the CYP2D6 and APOE genes or the ABCA1 and APOE genes (Lu et al., 2016; Lu et al., 2018), but more comprehensive clinical analyses of genes and corresponding in-depth studies of multiple genes are lacking. In addition, experimental studies of the mechanisms of action are needed. More studies on the relationship between multiple genes will lead to more accurate prediction of DNP clinical efficacy and are also conducive to the discovery of the pharmacological effects of DNP. There is a lack of studies on the combined impact of multiple genes on DNP efficacy. The mechanisms by which these genes affect DNP are largely unknown and merit further investigation.

There were limitations of the above studies, and the following are some points that should be included in future studies: (1) a long period for observation (more than 12 months); (2) a more specific focus, such as a focus specifically on the DNP efficacy in different stages of AD; (3) larger sample sizes; and (4) regulation of ethical and social issues. Numerous studies are needed before DNP treatment can be successfully translated into the clinic.

The reasons for the limited role of genomics in the clinical efficacy of DNP may be as follows: First, at present, the research on the clinical efficacy of DNP is mostly focused on the study of single gene, and the lack of comprehensive analysis of multiple genes, which is one of the reasons why the conclusions of these studies have limited clinical hints. Second, among the influencing factors of DNP's drug efficacy, what is the proportion of genes, this is also an unresolved question, and it is worth further research. However, several biomarkers might be promising to assess the treatment response of DNP. There is no doubt that genomics has absolutely important implications for the clinical efficacy of drugs. Some other studies suggest the significance of genomics for clinical treatment, such as VKORC1 (−1639G/A) and CYP2C9 (1075A/C) SNP, which greatly affect the clinical efficacy of warfarin (Zhenghong Qin, 2010); this conclusion has been very mature. At present, as far as we know, there have been research and development of related gene kits, which can be better applied to personalized medicine of warfarin.

In conclusion, the results of future studies will provide possible strategies for the evaluation of the DNP clinical efficacy and will also be beneficial for understanding the multiple mechanisms by which DNP produced a therapeutic effect in AD.

## Author Contributions

JL and XW were responsible for writing the manuscript, JL, LW, JF, and YZ were responsible for the literature review, YH was responsible for critical revision of the manuscript, CG was responsible for the manuscript review.

## Funding

This work was supported by a grant from the National Natural Science Foundation of China (grant No. 81202599); Shanghai Jiao Tong University Affiliated Sixth People's Hospital Science Foundation grant (No. ynlc201826); and a grant from Shanghai Municipal Health Commission (No.20194Y0052).

## Conflict of Interest

The authors declare that the research was conducted in the absence of any commercial or financial relationships that could be construed as a potential conflict of interest.
